# Supplementary material for: The New Nematicide Cyclobutrifluram Targets the Mitochondrial Succinate Dehydrogenase Complex in Caenorhabditis elegans
Source: J Dev Biol. 2023 Oct 19;11(4):39. doi: 10.3390/jdb11040039 (PMC10594496; doi:10.3390/jdb11040039)
Supplement: Supplementary file 1 [file jdb-11-00039-s001.zip › Supplementary Figure S1.pdf]

Supplementary Figure S1

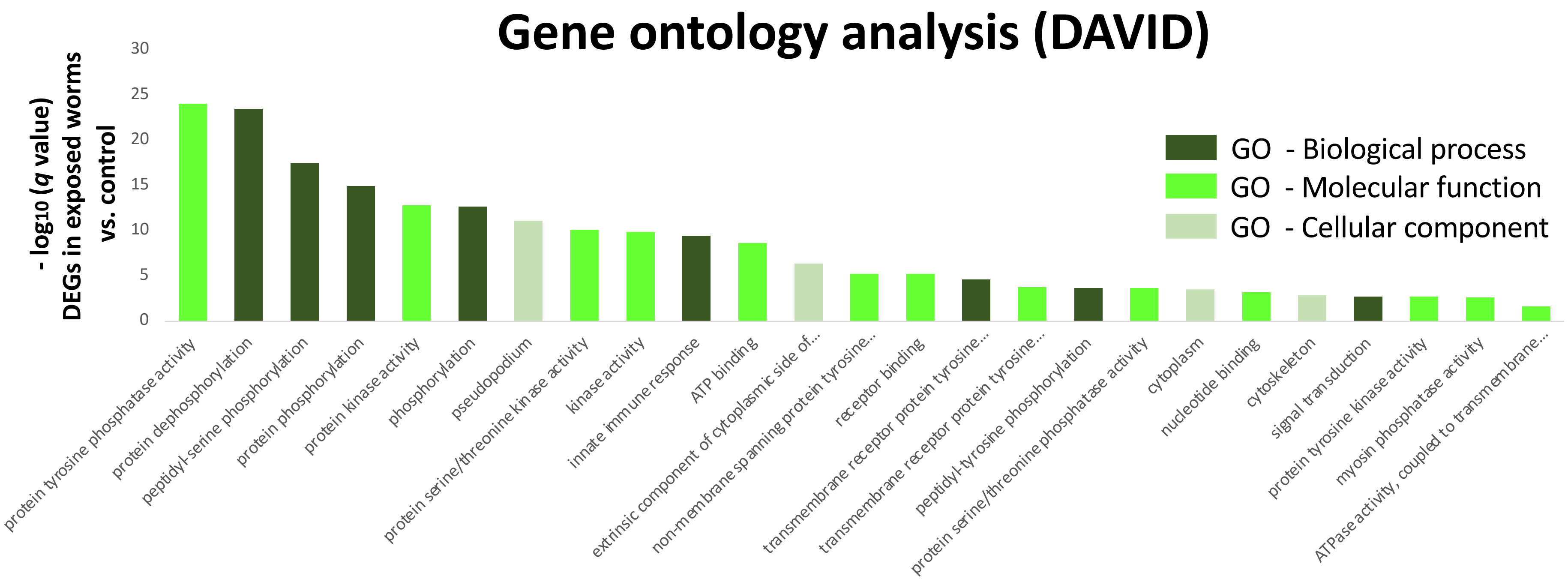

Figure S1: Histogram shows DAVID GO term functional analysis [33] (Huang et al.,2009) for DEGs in animals exposed to cyclobutrifluram compared with control non-exposed animals. Data are plotted as vertical bars that represent the significance of each GO-term. Adj. *P*-values (*q* values) were all significant (*q* value ≤ 0.05) and correspond to Benjamini–Hochberg correction.
